# Rapid on-site evaluation of touch imprints of medical thoracoscopy biopsy tissue for the management of pleural disease

**DOI:** 10.3389/fmed.2023.1196000

**Published:** 2023-06-09

**Authors:** Hansheng Wang, Yan Liu, Jiankun Wang, Tao Ren, Guoshi Luo, Hui You, Xiao Wang, Dan Li, Lei Wang, Meifang Wang

**Affiliations:** ^1^Department of Pulmonary and Critical Care Medicine, Taihe Hospital, Hubei University of Medicine, Shiyan, Hubei, China; ^2^Department of Pathology, Taihe Hospital, Hubei University of Medicine, Shiyan, Hubei, China; ^3^Department of Laboratory, Taihe Hospital, Hubei University of Medicine, Shiyan, Hubei, China

**Keywords:** malignant pleural effusion, medical thoracoscopy (MT), rapid on-site evaluation of cytology (ROSE), histopathology, biopsy, diagnosis, tuberculous pleural effusion (TPE)

## Abstract

**Background and objective:**

Medical thoracoscopy (MT) plays an important role in the diagnosis and treatment of pleural diseases, and rapid on-site evaluation (ROSE) has long been used for transbronchial needle aspiration or fine-needle aspiration to evaluate the adequacy of biopsy materials for the diagnosis of peripheral lung lesions. However, research on ROSE combined with MT for the management of pleural disease has been rarely reported. We aimed to evaluate the diagnostic performance of ROSE for pleura biopsies and visual diagnosis by thoracoscopists for gross thoracoscopic appearance. The secondary objective was to assess the intermodality agreement between ROSE and the final histopathologic diagnosis.

**Methods:**

A total of 579 patients with exudative pleural effusion (EPE) who underwent MT combined with ROSE from February 2017 to December 2020 at Taihe Hospital were included in the study. Thoracoscopists' visual diagnosis of gross thoracoscopic appearance, ROSE results, histopathologic findings, and the final diagnosis was recorded.

**Results:**

Thoracoscopic pleural biopsies were performed in 565 patients (97.6%); 183 patients were confirmed to have malignant pleural effusion (MPE), and 382 patients were confirmed to have benign pleural effusion (BPE). The area under the curve of ROSE for the diagnosis of MPE was 0.96 (95% CI: 0.94–0.98, *p* < 0.001), with a sensitivity of 98.7%, a specificity of 97.2%, a diagnostic accuracy of 97.1%, a positive predictive value of 97.2%, and a negative predictive value of 97.2%. Diagnostic consistency between ROSE and histopathology was good (κ ± SE = 0.93 ± 0.02, *p* < 0.001). The area under the curve of the thoracoscopists' visual diagnosis of gross thoracoscopic appearance was 0.79 (95% CI: 0.75–0.83, *p* < 0.01), with a sensitivity of 76.7%, a specificity of 80.9%, a positive predictive value of 62.4%, and a negative predictive value of 89.3%.

**Conclusion:**

ROSE of touch imprints of MT biopsy tissue during MT showed high accuracy for distinguishing between benign and malignant lesions. In addition, ROSE was in good agreement with the histopathological diagnosis, which may help thoracoscopists perform pleurodesis (talc poudrage) directly during the procedure, especially in patients with malignant results.

## Introduction

Pleural effusion is an abnormal accumulation of fluid in the pleural cavity (1), which is a common clinical symptom caused by cancer, tuberculous pleurisy, inflammation, and dysfunction of the heart, liver, kidney, and other organs (2). Currently, medical thoracoscopy (MT), a minimally invasive procedure that is efficient, safe, simple, and cost-effective, has distinct advantages in diagnosing and treating pleural effusion and pleural diseases (3, 4).

Rapid on-site evaluation (ROSE) is a cytomorphological diagnostic procedure that assesses the adequacy and accuracy of the material obtained during bronchoscopy within a few minutes in or near the bronchoscopy suite (on-site) using rapid staining (e.g., Diff-Quik or Hemacolor) of touch imprints of biopsies (5). ROSE has been reported to improve the diagnostic yield of transbronchial biopsy for endoscopically non-visible malignancy during transbronchial forceps biopsy (TFB) (6), to obtain high diagnostic performance and an acceptable rate of complications during computed tomography (CT)-guided fine-needle aspiration (FNA) for pulmonary lesions (7, 8), to significantly improve diagnostic yields (9), and to lower the additional number of biopsies (10–12) during transbronchial needle aspiration (TBNA).

However, there are few studies on ROSE of touch imprints of MT biopsy tissue for the management of pleural disease. Therefore, this study aimed to investigate the diagnostic performance of ROSE, including thoracoscopists' visual diagnosis of gross thoracoscopic appearance during the MT procedure. The secondary objective was to assess the consistency between ROSE results and histopathological diagnosis.

## Patients and methods

**Figure 1:**
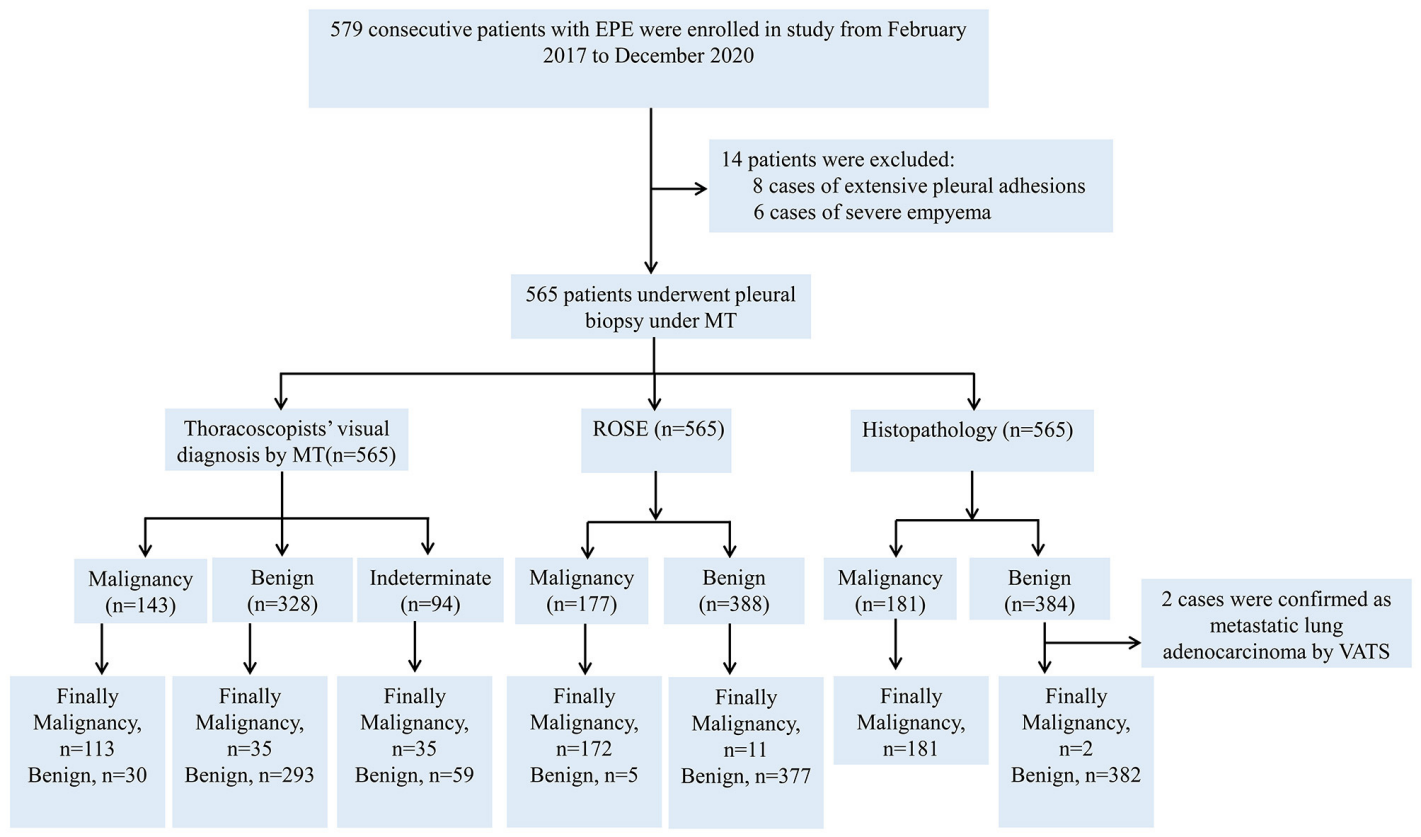
Flowchart for the selection and diagnosis of the study population.

A total of 579 consecutive patients were admitted due to EPE from February 2017 to December 2020 in Taihe Hospital and successfully underwent MT after evaluation for participation in the study. The inclusion criteria were as follows: (1) age above 18 years and (2) capable of undergoing MT. The exclusion criteria were as follows: (1) patients with contraindications for MT (13, 14) and (2) patients who did not provide written informed consent. The study flowchart is shown in Figure 1. The study was approved by the Ethics Committee of Taihe Hospital, and written informed consent was obtained from all patients. Patients underwent MT (LFT-240; Olympus, Tokyo, Japan) in the bronchoscopy room under conscious sedation with local anesthesia. Radiological evaluation was performed by chest CT, including the location of pleural effusion, amount of effusion, and any other abnormalities. Thoracic ultrasound (DP-20, Mindray, Shenzhen, China) was used to assess the exact location of the effusion, lacunae, and adhesions and the most appropriate entry site for thoracentesis. The extracted pleural fluid was submitted for biochemical, microbiological, and cytological examinations. Electrocardiogram, vital signs, and blood oxygen saturation were continuously monitored throughout the process. The patient was instructed to lie in a lateral decubitus position, breathing spontaneously with normal lungs in an independent position, with arms raised overhead. The marked skin surface on the affected side was thoroughly sterilized, and then 15–30 ml of 2% lidocaine was used to infiltrate the anesthetized chest wall to all layers of the pleura. A 1 cm incision was made in the midaxillary line between the fourth and seventh intercostal spaces in the chest wall, a trocar was inserted, and the pleural cavity was opened to atmospheric pressure. The pleural cavity was carefully examined, and any remaining pleural fluid was aspirated. Visual findings on gross thoracoscopic appearance were recorded, and a parietal pleural biopsy specimen was obtained under direct vision. A forceps biopsy was performed with forceps (FB-55CR-1 or FB-55KR-1, Olympus, Tokyo, Japan) to collect multiple (5–7) biopsy samples, and the number of biopsies obtained was recorded. At the end of the procedure, a chest tube was inserted, and lung dilation was confirmed by radiological examination before the chest tube was removed. Patients with complicated parapneumonic pleural effusions (CPEs)/empyema (frank pus) were implanted with a chest drain under thoracoscopic guidance (15). During MT, fluid and fibrinopurulent materials were aspirated, adhesiolysis was performed, biopsies were obtained, and a chest tube drain was inserted into the pleural space (15). According to the current guidelines, all patients with CPEs were treated with antibiotics. Chest radiographs were taken within 24 h, and patients were closely observed after thoracoscopy. The pleural tissue was immediately smeared or imprinted on a glass slide, stained with Diff-Quik, and interpreted or preliminarily diagnosed at the bedside if a cytopathologist was available on-site. The remaining pleural tissue was fixed with formalin and sent to the pathology department for hematoxylin and eosin (H&E) staining or auxiliary detection, such as immunohistochemical (IHC) and molecular tests. The thoracoscopic visual impression of the thoracoscopists and the cytological morphologic interpretation of the cytopathologists did not affect each other's diagnosis. Visual findings on gross thoracoscopic appearance and ROSE were interpreted by two thoracoscopists and two cytopathologists, respectively.

ROSE interpretation was reported as follows: (1) malignancy (e.g., the cytological characteristics of malignancy are demonstrated, and the cytological subtypes can be accurately categorized) that is specific to cytological subtypes; (2) suspicious for malignancy (e.g., some abnormal cells are found, which cannot be confirmed as cancer cells); and (3) negative for malignancy (e.g., ROSE specimens revealed granulomas, reactive mesothelial, or acute/chronic non-specific inflammatory cells or others). We categorized the cytological subtypes of malignancy in accordance with the International Association for the Study of Lung Cancer (IASLC)/American Thoracic Society (ATS)/European Respiratory Society (ERS) (16).

The final diagnosis was made based on the histological and clinical/microbiological findings. Formalin-fixed paraffin-embedded (FFPE) pleural tissues were examined by two experienced pathologists in a double-blind manner after H&E or IHC staining. IASLC/ATS/ERS guidelines were used as the reference standard for the histological subtype classification of malignancy (16). Ziehl–Neelsen (Z–N) staining, nucleic acid amplification test (NAAT), and cultures were performed on the corresponding biopsy specimens to confirm or exclude the diagnosis of tuberculosis in cases with histopathological suspicion of tuberculosis. Z–N staining was performed according to the standard protocols (17), and the results were graded according to the American Thoracic Society/Centers for Disease Control and Prevention guidelines (18). NAAT was performed according to the manufacturer's instructions (19, 20) under the following amplification conditions: 37°C for 5 min, followed by 40 cycles of 94°C for 1 min, 95°C for 5 s, and 60°C for 30 s. The reaction system volume was 40 μl. In this study, the gold standard for the diagnosis of tuberculous pleurisy was the presence of the following: (1) positive Z–N staining or culture in pleural biopsy; (2) caseous granuloma in pleural biopsy; (3) positive NAAT on pleural biopsy and favorable response to anti-tuberculous medications; and (4) pleural biopsy with non-caseating epithelioid granulomas, no other causes of non-caseating granulomas, and favorable response to anti-tuberculous medications. All patients were followed up for at least 12 months after MT. After histopathological examination, patients diagnosed with non-specific pleurisy (NSP) who were not candidates for thoracoscopic surgery were further investigated with video-assisted thoracoscopic surgery (VATS) for persistent or recurrent pleural effusion (4). According to the final diagnosis, the enrolled patients were divided into two groups: Group A included patients with malignancy, and Group B included patients with non-malignancy.

### Statistical analysis

SPSS software version 26.0 was used for statistical analysis. Measurement data with a normal distribution are expressed as the mean ± standard deviation (mean ± SD), and a *t*-test was used. Measurement data with a non-normal distribution are expressed as the median and interquartile range, and the Mann–Whitney *U*-test was used. The chi-square test or Fisher's exact test was used to compare the categorical data. Receiver operator characteristic (ROC) curves were designed to assess sensitivity, specificity, positive predictive values (PPVs), and negative predictive values (NPVs) for the estimated parameters. A chi-square test was used to compare diagnostic accuracy rates between the disease-specific groups. Consistency between ROSE results and final histopathological diagnosis was assessed by calculating a κ score. Probability values < 5% (*p* < 0.05) were considered statistically significant.

## Results

**Figure 2:**
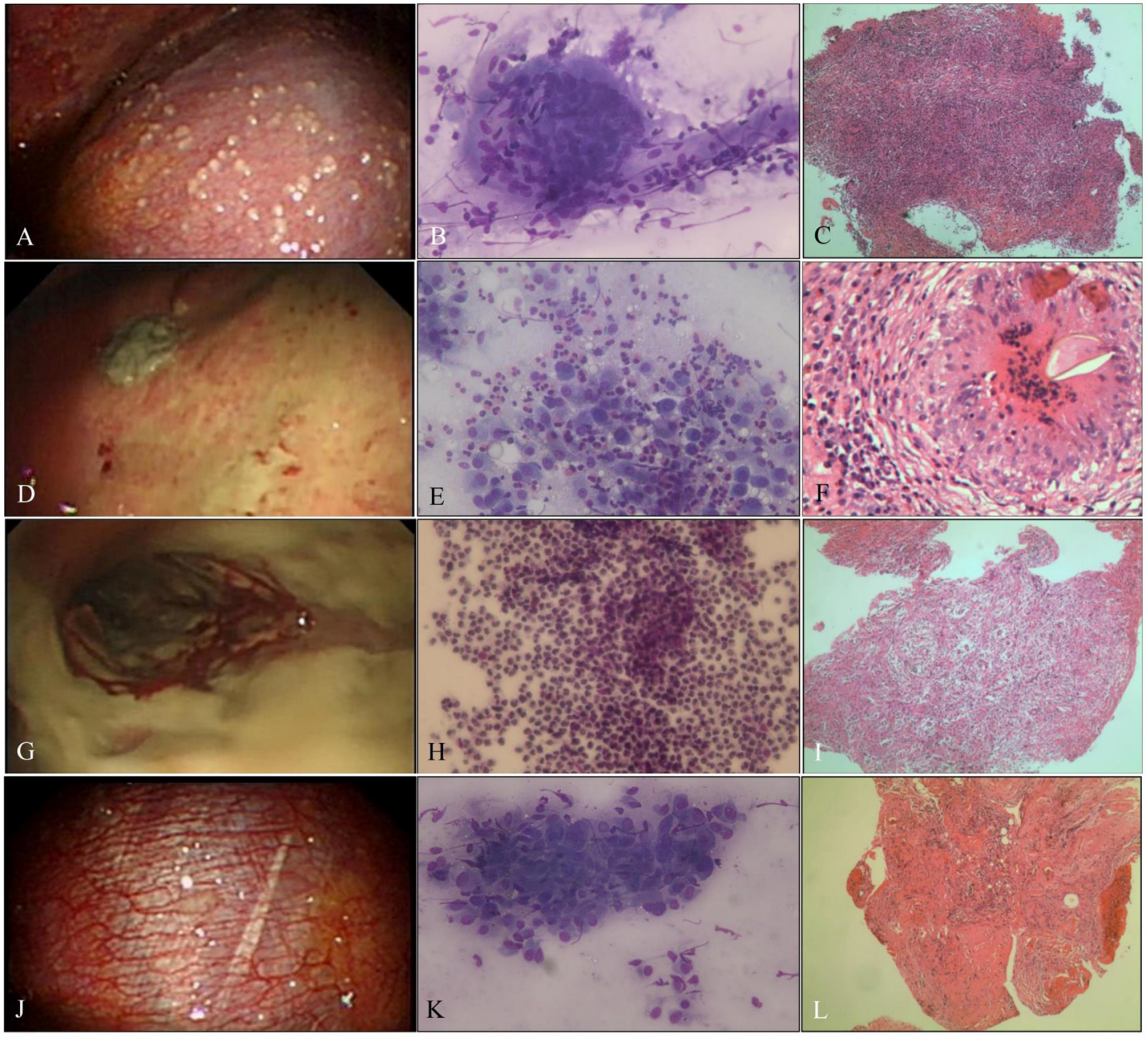
Benign pleural disease. **(A)** Tuberculous pleurisy with sago-like nodules on the parietal pleura. **(B)** ROSE of touch imprints of pleural biopsies shows epithelioid cell granulomas with necrosis, and the diagnosis was tuberculosis (Diff-Quik stain, × 400). **(C)** Necrotizing granuloma with positive NAAT and positive Z–N staining; tuberculosis was diagnosed (H&E stain, × 200). **(D)** Indeterminate thoracoscopic appearance, yellow-white necrosis on the pleura. **(E)** ROSE of touch imprints of the yellow-white necrotic biopsies show eosinophils, and the diagnosis was NSP (Diff-Quik stain, × 400). **(F)** Paragonimiasis, eggs, and structure of paragonimiasis were found in the granulomas (H&E stain, × 400). **(G)** Empyema with purulent pleural moss on the pleura. **(H)** ROSE of touch imprints of purulent pleural moss revealed a full field of neutrophils (Diff-Quik stain, × 400). **(I)** Acute and chronic inflammatory cell infiltration with cellulosic exudation (H&E stain, × 200). **(J)** NSP, hyperemic, and thickened pleura without fibrinogenic adhesions. **(K)** Inflammation, ROSE of touch imprints of pleural biopsies interpreted as fibrocyte/mesothelial hyperplasia with chronic inflammatory cells (Diff-Quik stain, × 400). **(L)** Non-specific inflammation, fibrous tissue hyperplasia, and mesothelial hyperplasia with fibrinous exudation (H&E stain, × 200).

**Figure 3:**
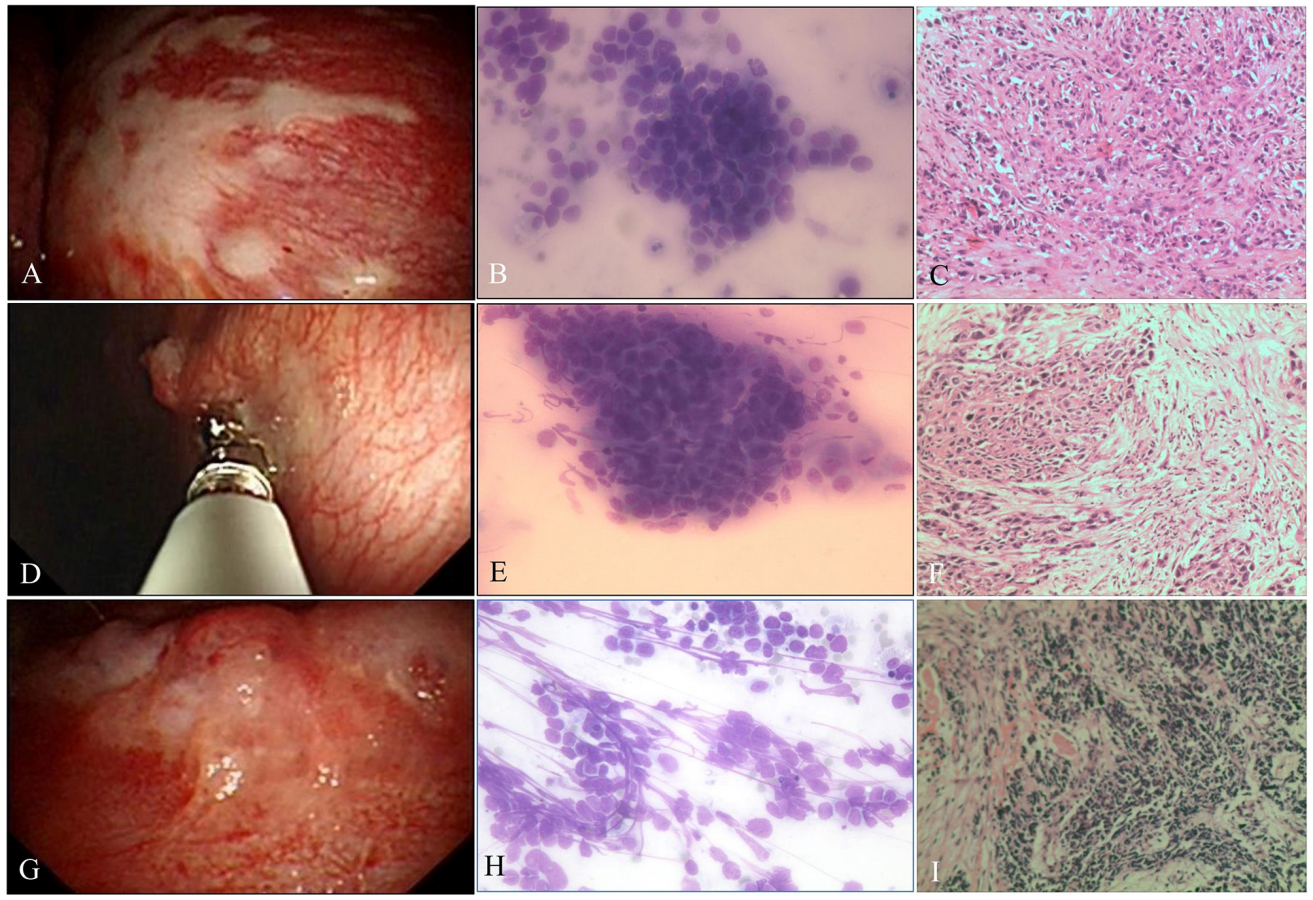
Malignant pleural disease. **(A)** Pleural plaque-like lesions. **(B)** Poorly differentiated AdC. ROSE shows dyscohesive aggregate cells with large nuclei, prominent nucleoli, and tumor cells with single intracytoplasmic vacuoles or globular secretory material, which indicate glandular differentiation (Diff-Quik stain, × 400). **(C)** Metastatic lung AdC, where the tumor is poorly differentiated and has obvious atypia, some cancer cells are arranged singly, and the mitotic image is easily seen (H&E stain, × 200). **(D)** Malignancy, pleural nodules. **(E)** Well-differentiated SqCC, with smears composed of mainly dispersed, often elongated or spindle-shaped cells with dense cytoplasm and keratinization. The nuclei are often pyknotic or hyperchromatic with angulated contours (Diff-Quik stain, × 400). **(F)** Metastatic lung SqCC. The cancer cells showed solid arrangement, lack of keratinization and interbridging, light pink cytoplasm, and obvious atypia (H&E stain, × 200). **(G)** Malignancy, nodules fused into masses. **(H)** SCC. ROSE shows small cells with a high N/C ratio, cells arranged like a mosaic or spinal cord, “salt and pepper” chromatin texture, and nuclear molding, which are consistent with small cell carcinoma (Diff-Quik stain, × 400). **(I)** Metastatic lung SCC. The tumor cells are closely arranged in sheets, with round or oval nuclei, fine granular chromatin, no obvious nucleoli, sparse cytoplasm, and mitotic images (H&E stain, × 200).

**Figure 4:**
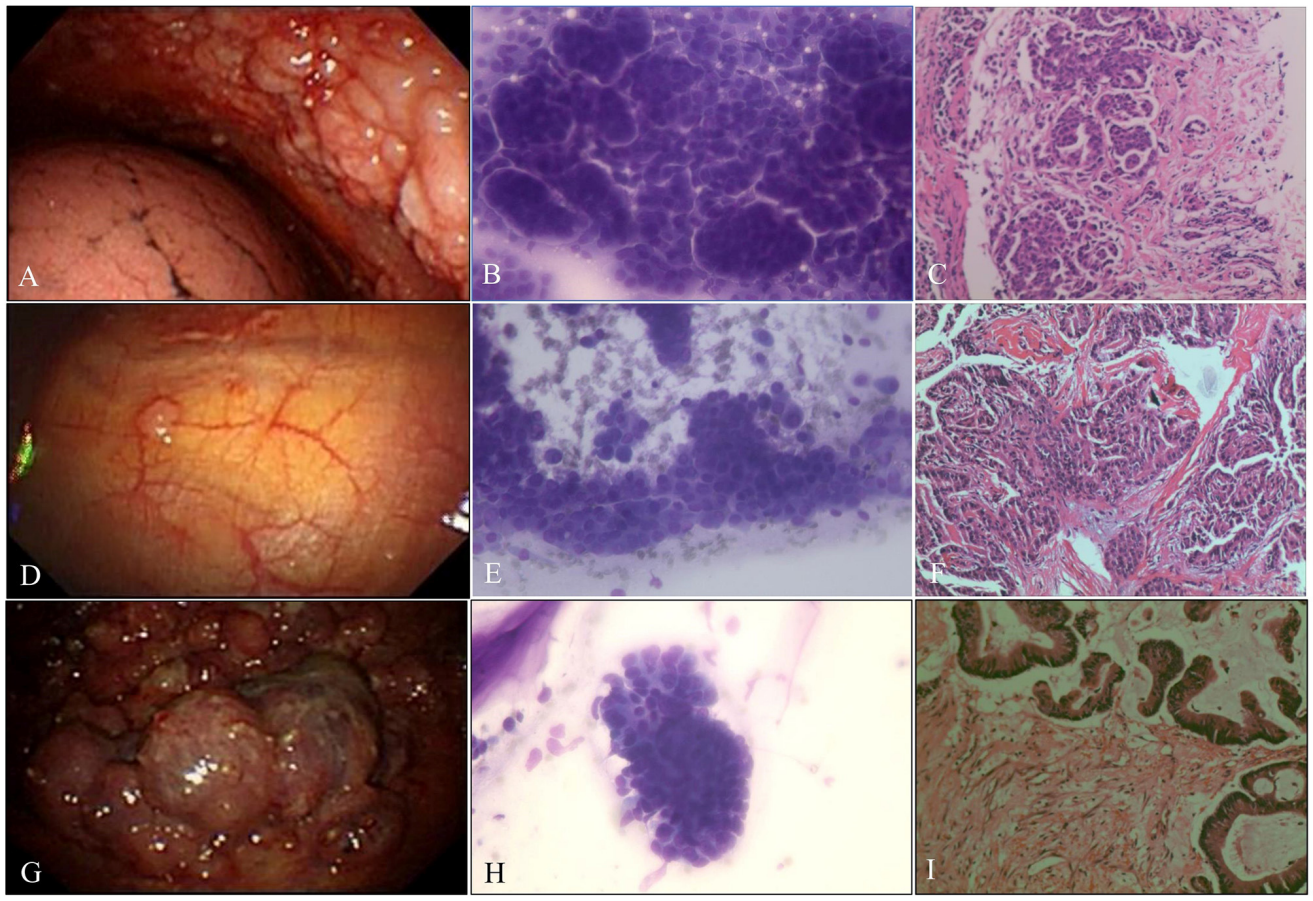
Malignant pleural disease. **(A)** Malignancy with diffuse nodules. **(B)** AdC. ROSE shows cancer cells arranged in clumps or mulberry-like (Diff-Quik stain, × 400). **(C)** Metastatic breast cancer. The tumor cells were arranged in a nest-like pattern (H&E stain, × 200). **(D)** Indeterminate, single, scattered nodule. **(E)** AdC. ROSE shows papillary architecture; enlarged, crowded, and often molded nuclei; and fine nuclear chromatin (Diff-Quik stain, × 400). **(F)** Metastatic papillary thyroid carcinoma. The tumor cells are arranged in a papillary manner, the tumor cells are large, and the nucleoli are obvious (H&E stain, × 200). **(G)** Malignancy, cauliflower-like neoplasm. **(H)** AdC. ROSE shows cancer cells arranged in high columns with a high N/C ratio and obvious nucleoli (Diff-Quik stain, × 400). **(I)** Metastatic colon cancer. High columnar cancer cells were arranged in a glandular pattern with eosinophilic cytoplasm and brush borders on the free surface (H&E stain, × 200).

**Figure 5:**
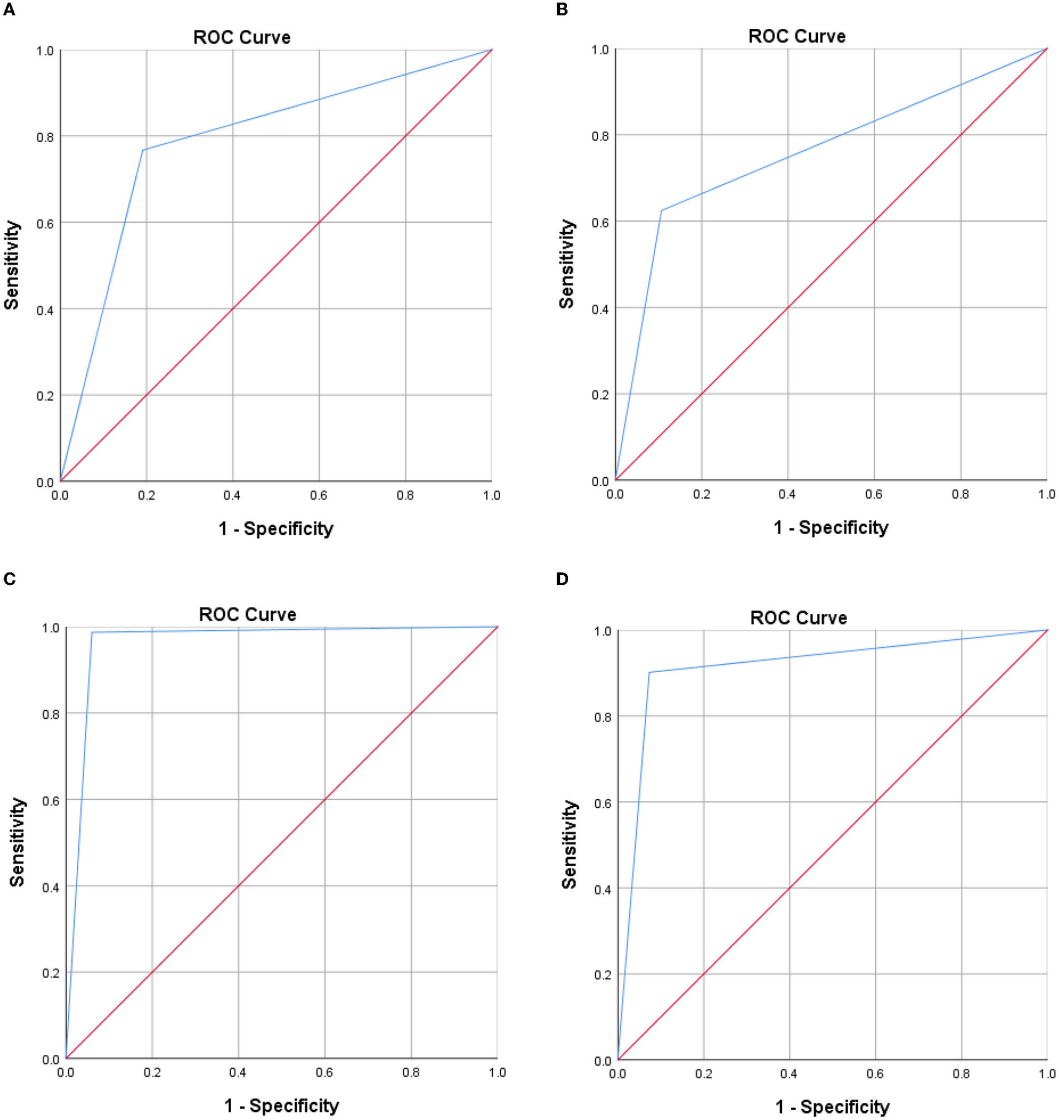
Receiver operator characteristic curve. **(A)** Thoracoscopists' visual diagnosis of malignant pleural diseases. **(B)** Thoracoscopists' visual diagnosis of benign pleural diseases. **(C)** ROSE in the diagnosis of malignant pleural diseases. **(D)** ROSE in the diagnosis of tuberculous pleural diseases.

A total of 579 patients were evaluated for participation in the study; eight cases of extensive pleural adhesions and six cases of severe empyema, resulting in no pleural space for biopsy, were excluded. The flowchart for the selection and diagnosis of the study population is shown in Figure 1. Ultimately, 565 patients (97.6%) were diagnosed by pleural biopsy. Group A consisted of 183 patients with malignant pleural disease, and Group B consisted of 382 patients with benign pleural disease. Demographic characteristics, clinical presentation, radiological findings, characteristics of pleural fluid, and final diagnosis are summarized in Table 1; laboratory results of pleural fluid are detailed in Table 2; and counts of peripheral blood cells and coagulation function are shown in the Supplementary Table. CT findings of pleural thickening and pleural nodularity had a certain predictive effect in malignant pleural diseases (*p* < 0.01 and *p* < 0.01, respectively). The appearance of yellow pleural fluid had a certain predictive value for benign pleural diseases (*p* < 0.01), while the appearance of bloody pleural fluid had a certain predictive value for malignant pleural diseases (*p* < 0.01). According to the Light criteria (21), the number of patients with exudative pleural effusion in Group A and Group B was 178 and 366, respectively, and no significant difference was observed (*p* = 0.39). As shown in Table 3, we observed that there were significantly more patients with hyperemic and thickened pleura, pleural masses, and pleural plaque-like lesions of thoracoscopic appearance in Group A than in Group B; in other words, these thoracoscopic findings have the potential to predict malignant pleural diseases (**Figures 3**, **4**) (*p* = 0.03, *p* < 0.01, and *p* < 0.01, respectively), while fibrinogenic adhesions, encapsulated effusion, purulent pleural moss, and caseous necrosis appeared to be predictive of benign pleural disease (Figure 2) (*p* < 0.01, *p* = 0.002, *p* = 0.018, and *p* < 0.01, respectively). The thoracoscopists' visual diagnosis of macroscopic appearance under MT is detailed in Table 3. The receiver operator characteristic (ROC) curve for the diagnosis of malignant pleural diseases is shown in **Figure 5A**, with an area under the curve (AUC) of 0.79 (95% CI: 0.75–0.83, *p* < 0.001), a sensitivity of 76.7%, a specificity of 80.9%, a PPV of 62.4%, and an NPV of 89.3%. The ROC curve for the diagnosis of benign pleural diseases is shown in **Figure 5B**, with an AUC of 0.76 (95% CI: 0.72–0.80, *p* < 0.001), a sensitivity of 62.4%, a specificity of 89.3%, a PPV of 76.7%, and an NPV of 80.9%.

**Table 1:** Demographic characteristics, clinical presentation and final diagnosis in enrolled patients (*n* = 565).

**Characteristics**	**Group A (*n* = 183)**	**Group B (*n* = 382)**	**χ^2^/t**	***p*-value**
Age (years, mean ± SD)	62.1 ± 12.0 (18–89)	47.9 ± 17.1 (19–87)	59.3	0.00
Gender (male/female), *n*	96/87	266/116	15.85	0.00
**Smoking history**				
Never smoker, *n* (%)	97 (53.0)	173 (45.2)	2.95	0.09
Ex-smoker, *n* (%)	58 (31.7)	113 (29.5)	0.26	0.61
Current smoker, *n* (%)	28 (15.3)	96 (25.3)	6.98	0.01
**Clinical symptoms**				
Cough, *n* (%)	152 (83.1)	265 (69.3)	11.99	0.001
Expectoration, *n* (%)	117 (64.2)	191 (50.0)	9.69	0.002
Chest pain, *n* (%)	124 (68.0)	247 (64.7)	0.527	0.468
Dyspnea, *n* (%)	146 (79.7)	163 (42.8)	68.77	< 0.01
Fever, *n* (%)	24 (13.3)	104 (27.4)	14.06	< 0.01
Weight loss, *n* (%)	139 (75.8)	151 (39.5)	65.72	< 0.01
Night sweats, *n* (%)	2 (1.1)	58 (15.3)	25.88	< 0.01
Fatigue, *n* (%)	103 (56.5)	139 (36.5)	20.00	< 0.01
Hemoptysis, *n* (%)	0 (0)	12 (3.2)	5.87	0.015
**CT imaging**, ***n*** **(%)**				
Pleural thickening	146 (79.8)	186 (48.7)	49.35	< 0.01
Atelectasis	133 (72.5)	260 (68.1)	1.24	0.265
Pleural nodularity	102 (56.0)	13 (3.5)	209.04	< 0.01
**Side of pleural effusion**				
Right, *n* (%)	96 (52.5)	191 (50)	0.299	0.584
Left, *n* (%)	83 (45.4)	174 (45.5)	0.002	0.965
Bilateral, *n* (%)	4 (2.1)	17 (4.5)	1.773	0.183
**Amount of pleural effusion**				
Mild, *n* (%)	34 (18.6)	92 (24.1)	2.164	0.141
Moderate, *n* (%)	36 (19.7)	76 (19.9)	0.004	0.950
Massive, *n* (%)	113 (61.7)	214 (56.0)	1.665	0.197
**Appearance of pleural fluid**				
Yellow	109 (59.6)	339 (88.7)	64.163	< 0.01
Bloody/blood-tinged	66 (36.1)	14 (3.7)	106.87	< 0.01
Purulent	0 (0)	15 (3.9)	7.382	0.007
Other	8 (4.3)	14 (3.7)	0.165	0.684
**Diagnosis**, ***n*** **(%)**				
	Metastatic lung cancer 143 (78.1)	Tuberculosis 301 (78.8)		
	Metastatic extrathoracic cancer 28 (15.3)	Parapneumonic effusion 31 (8.0)^▴^		
	Malignant mesothelioma 8 (4.4)	Non-specific pleuritis 47 (12.3)^Δ^		
	Lymphoma 4 (2.2)	Paragonimiasis 3 (0.79%)		

^▴^Including five cases of complicated parapneumonic effusion (CPPE), nine cases of uncomplicated parapneumonic effusion (UPPE), 17 cases of empyema.

^Δ^Forty-seven patients diagnosed with non-specific pleuritis (NSP) were followed up for up to 24 months. a spontaneous resolution of the effusion occurred in 33 cases, pleural effusion associated with systemic lupus erythematosus (SLE) in three cases, associated with rheumatoid arthritis in two cases, the remaining nine patients were diagnosed with idiopathic pleuritis.

**Table 2:** Laboratory results of pleural fluid in enrolled patients (*n* = 565).

**Laboratory tests**	**Group A (*n* = 183)**	**Group (*n* = 382)**	**χ^2^/*t*/Z**	***p*-value**
**Pleural fluid**				
Light criteria (exudate/transudates), *n*^#^	178/5	366/16	0.73	0.39
Nucleated cell counts, 10^6^/L	2,405.0 (1,323.0, 3,610.0)	90,778.0 (5,167.0, 362,095.0)	−509.7	< 0.01
Percentage of monocyte, %	90.0 (80.0, 94.0)	95.0 (80.0, 94.0)	−67.3	< 0.01
Percentage of multinucleated cell, %	42.0 (19.0, 68.0)	48.0 (15.0, 80.0)	−5.8	< 0.01
ADA, U/L	20.5 (13.4, 110.4)	74.2 (57.9, 97.2)	−46.7	< 0.01
LDH, U/L	1,052.0 (484.0, 3,235.0)	845.4 (510.0, 3,745.0)	−3.2	0.001
Hs-CRP, mg/L	24.6 (14.3, 34.5)	44.1 (29.2, 70.4)	−34.8	< 0.01
Total cholesterol, mmol/L	2.2 (1.83, 2.55)	2.3 (1.95, 2.72)	−3.78	< 0.01
Amylase, U/L	288.1 (93.4, 556.6)	43.0 (35.0, 54.8)	−125.6	< 0.01
CEA, μg/L	1,000 (591.1, 2,332.0)	6.22 (5.2, 7.7)	−80.4	< 0.01

^#^Chi-square test used; others are Mann-Whitney *U*-test.

**Table 3:** Thoracoscopic findings, thoracoscopist's visual diagnosis under MT in enrolled patients (*n* = 565).

**Findings/diagnosis**	**Group A (*n* = 183)**	**Group B (*n* = 382)**	**χ^2^/t**	***p*-value**
**Thoracoscopic findings**				
Hyperemic or thickened pleura, *n* (%)	177 (96.7)	351 (91.9)	4.729	0.03
Pleura nodules, *n* (%)	114 (62.3)	231 (60.5)	0.173	0.677
Pleural masses, *n* (%)	2 (1.1)	0	538.03	< 0.01
Fibrinogenic adhesions, *n* (%)	81 (44.3)	305 (79.8)	72.369	< 0.01
Encapsulated effusion, *n* (%)	4 (2.2)	35 (9.2)	9.371	0.002
Purulent pleural moss, *n* (%)	1 (0.5)	16 (4.2)	5.624	0.018
Pleural caseous necrosis, *n* (%)	1 (0.5)	182 (47.6)	125.327	< 0.01
Pleural plaque-like lesions, *n* (%)	37 (20.2)	3 (0.8)	71.029	< 0.01
**Thoracoscopist's visual diagnosis of macroscopic appearance under MT**				
Malignant, *n* (%)	99 (54.1)	44 (11.5)		
Benign, *n* (%)	35 (19.1)	293 (76.7)		
Indeterminate, *n* (%)	49 (26.8)	45 (11.8)		
Total pleural biopsies (mean ± SD)	5.95 ± 1.05	5.96 ± 0.85	1.31	0.19
ROSE diagnostic samples (mean ± SD)	2.94 ± 0.91	3.06 ± 0.74	1.84	0.07

The average numbers of pleural biopsies in Groups A and B under MT were 5.95 ± 1.05 (mean ± SD) and 5.96 ± 0.85 (mean ± SD), respectively, and no significant difference was observed. The ROSE diagnostic samples of the two groups were 2.94 ± 0.91 (mean ± SD) and 3.06 ± 0.74 (mean ± SD), respectively, and there was no significant difference, as detailed in Table 3. As shown in Table 4, of the 183 pleural samples with a final diagnosis of malignancy (Figures 3, 4), three cases were diagnosed as tuberculosis, and eight cases were diagnosed as inflammatory by ROSE. In 301 cases with a final diagnosis of tuberculosis in pleural samples (Figure 2), ROSE was interpreted as malignancy in five cases and inflammation in 17 cases. Among 78 cases finally diagnosed as inflammatory, 31 cases were parapneumonic effusion and 47 cases were non-specific pleuritis, of which 21 cases were interpreted as tuberculosis by ROSE. In two of the three cases of benign pleural diseases associated with paragonimiasis infection, ROSE misdiagnosed one as tuberculosis, and the other case was interpreted as granulomatous inflammation with eosinophils. As a result, the sensitivity, specificity, diagnostic accuracy, PPV, and NPV of ROSE in the diagnosis of malignant pleural diseases with pleura tissues were 98.7, 92.3, 97.1, 97.2, and 97.1%, respectively, with an AUC of 0.963 (95% CI: 0.942–0.984, *p* < 0.001), as shown in Figure 5C. The sensitivity, specificity, diagnostic accuracy, PPV, NPV, and AUC (95% CI: 0.752–0.912, *p* < 0.001) of ROSE for the diagnosis of tuberculous pleural diseases were 90.2%, 92.7%, 91.5%, 91.5%, 91.5%, and 0.91 (95% CI: 0.887–0.941, *p* < 0.001) (Figure 5D), respectively. As shown in Table 5, the agreement between ROSE and histopathology in the morphological diagnosis of pleural biopsies of malignant pleural disease was as follows: there was very good agreement in the diagnosis of small cell carcinoma (κ ± SE = 0.938 ± 0.061, *p* < 0.001), good agreement in the diagnosis of adenocarcinoma (κ ± SE = 0.636 ± 0.069, *p* < 0.001), good agreement in the diagnosis of squamous cell carcinoma (κ ± SE = 0.719 ± 0.154, *p* < 0.001), and good agreement in the diagnosis of lymphoma (κ ± SE = 0.658 ± 0.185, *p* < 0.001); however, there was only general agreement in the diagnosis of malignant mesothelioma (κ ± SE = 0.534 ± 0.182, *p* < 0.001).

**Table 4:** Correlation between ROSE results and final diagnosis in the differential diagnosis of benign and malignant pleural diseases (*n* = 565).

**ROSE**	**Final diagnosis**				
	**Malignancy**	**Tuberculosis**	**Inflammatory**	**Paragonimiasis**	**Total**
Malignancy	172	5	0	0	177
Tuberculosis	3	279	21	2	305
Inflammatory	8	17	57	0	82
Paragonimiasis	0	0	0	1	1
Total	183	301	78	3	565

**Table 5:** Consistency between ROSE and histopathologic diagnosis of thoracoscopic pleural biopsies in patients with malignant pleural diseases (*n* = 183).

**ROSE**	**Histopathology**							
	**AdC**	**SqCC**	**SCC**	**Malignant mesothelioma**	**Lymphoma**	**Other malignancy**	**Suspicious malignancy**	**Non-malignancy**	**Total**
AdC	131	3	0	3	0	1		2	140
SqCC		4	0	0	0				4
SCC		0	8	0	0				8
Malignant mesothelioma		0	0	3	0				3
Lymphoma		0	0	0	3	2			5
Other malignancy		0	1	0	0				1
Suspicious malignancy	12					4			16
Non-malignancy	2	0	0	2	1	1			6
Total	145^†^	7^‡^	9	8	4	8^§^	0	2^*^	183

AdC, adenocarcinoma; SqCC, squamous cell carcinoma; SCC, small cell carcinoma.

^**†**^Including 123 cases of metastatic lung AdC, 10 cases of metastatic breast cancer, three cases of metastatic ovarian serous adenocarcinoma, five cases of metastatic gastric cancer, one case of metastatic colon cancer, one case of metastatic rectal cancer, one metastatic clear cell renal cell carcinoma, one metastatic thyroid cancer.

^**‡**^Including four cases of metastatic SqCC of the lung, three cases of metastatic SqCC of the esophagus.

^**§**^Including 8 cases of other metastatic malignancies, as follow, B3 thymoma, primitive neuroectodermal tumor (PNET), sclerosing epithelioid fibrosarcoma (SEF), extralrenal malignant rhabdoid tumor (EMRT), malignant melanoma, Sarcomatoid carcinoma, Adenoid cystic carcinoma, Ewing's sarcoma.

^*^The pleural biopsy specimens of these two cases were diagnosed as benign by histopathology, but highly suspected of malignancy by clinical and imaging, and subsequently confirmed as metastatic lung adenocarcinoma by VATS.

## Discussion

We conducted a retrospective cohort study with consecutive patients. Our study indicated that during the diagnosis of malignant pleural disease with pleural biopsies, ROSE showed a good sensitivity of 98.7% and a specificity of 93.2%, which was better than that of the thoracoscopists' visual diagnosis of macroscopic appearance under MT, whose sensitivity and specificity were 76.7 and 80.9%, respectively. In addition, the agreement between ROSE and histopathology in the morphological diagnosis of pleural biopsies of malignant pleural disease was good, especially for small cell carcinoma, adenocarcinoma, squamous cell carcinoma, and lymphoma.

Malignant pleural tumors are often accompanied by pleural effusion, which is rich in malignant cells (22). However, the sensitivity of pleural effusion cytology for the diagnosis of malignant tumors is only 60% (40.0–87.0%) (4, 23), and tissue sampling is usually needed. In the past, pleural biopsies performed with traditional biopsy needles with hook notches, such as Abrams or Cope, had a sensitivity of 40–74% for malignant pleural diseases (24). MT significantly improves the positive diagnostic rate of pleural diseases by comprehensively observing the pleural cavity and performing a biopsy on suspicious lesions under visual conditions (25). Studies have shown that the sensitivity of medical thoracoscopic biopsy in the diagnosis of MPE is in the range of 92.6–97% and the specificity is 99–100% (23). The thoracoscopic appearance of MPE mostly presented nodules of varying sizes, which could be grape-shaped or cauliflower-shaped. Some nodules fused into masses and presented hyperemic or thickened pleura, pleural adhesion or diffuse nodules, and a few showed plaque-like lesions. Our study found that the thoracoscopists' visual diagnosis for malignancy under MT showed a sensitivity of 76.7% and a specificity of 80.9%; however, Davies et al. (26) reported that the diagnostic sensitivity and specificity of the thoracoscopists' impression for distinguishing pleural malignancy were 100 and 21%, respectively, and the sensitivity was lower than the 100% they reported, possibly because the thoracoscopic appearance of some MPEs mimicked the thoracoscopic appearance of TPE in our larger population study. The thoracoscopic appearance of TPE in the acute stage mainly presents with parietal pleural hyperemia and edema, sago-like nodules, or scattered nodules. Chronic TPE is characterized by grayish-white and light yellow cellulose deposition with uneven thickness and encapsulated effusion formed by extensive adhesion. Previous studies demonstrated that the presence of sago-like nodules on gross thoracoscopic appearance is highly specific for TPE, with a sensitivity of 58.9%, a specificity of 92.6%, and a diagnostic accuracy of 69.88% (27, 28). Thomas et al. concluded that the presence of sago-like nodules on the gross thoracoscopic appearance of the pleural surface had a sensitivity of 58%, a specificity of 89%, and a diagnostic accuracy of 62% (29). Our study found that the thoracoscopists' visual diagnosis for benign nodules under MT showed a sensitivity of 62.4%, which is higher than previously reported, mainly because benign pleural effusion in our study included not only TPE but also NSP and parapneumonic effusion, thus leading to a higher sensitivity.

To date, only Porfyridis et al. (30) have reported the application of ROSE in MT biopsy, with an AUC of 0.86 (95% CI: 0.76–0.96, *p* < 0.001), a sensitivity of 79.17%, a specificity of 94.59%, a diagnostic accuracy of 88.5%, a PPV of 90.5%, and an NPV of 87.5% in the diagnosis of malignant pleural diseases. In contrast to their results, in our study, ROSE showed a better performance in the identification of malignant pleural diseases, with an AUC of 0.963 (95% CI: 0.942–0.984, *p* < 0.01), a sensitivity of 98.7%, a specificity of 92.3%, a diagnostic accuracy of 97.1%, a PPV of 97.2%, and an NPV of 97.1%. Compared with the diagnosis of malignant pleural disease, the diagnostic sensitivity (90.2%), diagnostic accuracy (91.5%), PPV (91.5%), and NPV (91.5%) of ROSE for pleural tuberculosis samples were lower. Interestingly, there were two patients who were initially diagnosed with the benign disease by histopathology, but ROSE was interpreted as adenocarcinoma, and they were finally confirmed as having metastatic lung adenocarcinoma by VATS. This may prove that ROSE has better diagnostic performance for malignant tumors, as Chandra et al. (31) reported that ROSE cytology and histology were comparable, and ROSE may be superior to histopathology for the diagnosis of lung tumors. Previous studies have proved that talc was the most effective and used agent for pleurodesis in patients with recurrent pleural effusions (32–36). Prior to performing thoracoscopic talc poudrage, it is important to understand etiology, especially recurrent pleural effusion with malignant etiology. In clinical practice, patients with already diagnosed MPEs can be submitted to pleurodesis by talc poudrage; or patients with recurrent pleural effusion presenting with a history of advanced malignant disease are suspected to have MPE and pleurodesis with talc poudrage can be performed; or by performing a VATS biopsy to investigate the cause of pleural effusion and provide adequate pleurodesis at the same time. Therefore, intraoperative diagnosis plays an important role in the direct implementation of thoracoscopic talc poudrage. In this sense, we suggest that this advantage of ROSE may help thoracoscopists directly perform pleurodesis (talc poudrage) on patients with MPE during the procedure. Tuberculosis is endemic in China (37). ROSE of touch imprints of pleural biopsies that are interpreted as epithelioid cell granulomas with scattered lymphocytes and tuberculous pleural disease then needs to be highly suspected. In addition, ROSE should be interpreted with caution in diagnosing tuberculous pleurisy because granulomatous pleurisy can occur in sarcoidosis, rheumatoid arthritis, histoplasmosis, Aspergillus disease, paragonimiasis infection, and other diseases (38). Among the patients included in our study, three were eventually diagnosed with pleural effusion caused by *paragonimiasis* infection, and their pleural specimens were interpreted as granulomatous with eosinophil infiltration by ROSE; however, the presence of eosinophils acts as a key point of differential diagnosis between *paragonimiasis* infection and tuberculous pleurisy (39). Morphologically, ROSE showed good agreement with histopathology in the diagnosis of malignant tumors, mainly some specific types of tumors, such as small cell carcinoma, adenocarcinoma, squamous cell carcinoma, and lymphoma, with kappa values of 0.938, 0.636, 0.719, and 0.658, respectively, Wang et al. (6) reported good agreement between ROSE and histology of transbronchial biopsies, with kappa values of 0.749, 0.728, and 0.940 for squamous cell carcinoma, adenocarcinoma, and small cell carcinoma diagnosis, respectively. Moreover, other studies demonstrated that ROSE and pathology were well-correlated with the diagnosis of squamous cell carcinoma (kappa = 0.718, *p* < 0.05), adenocarcinoma (kappa = 0.662; *p* < 0.05), and small cell lung cancer (kappa = 0.955; *p* < 0.05) during bronchoscopic biopsy. In general, our study is consistent with previous reports. Celik et al. concluded that ROSE can be used in underresourced laboratories and low-income countries without IHC (40). However, ROSE should be interpreted with caution. In our study, five pleura biopsies that were finally diagnosed as tuberculosis were misinterpreted by ROSE as malignant tumors, which were reactive mesothelial cells. The differential diagnosis between reactive mesothelial cells and malignant mesothelioma or adenocarcinoma cells may sometimes be challenging, and possible pitfalls for ROSE, e.g., ongoing lung/pleural repair and regeneration processes, might make the interpretation of on-site cytology more difficult for a non-pathologist, similar to what has been observed in different diseases (41, 42). In doubtful cases, IHC stains can confirm the mesothelial origin; calretinin, WT-1, HBME-1, and D2-40 are positive in mesothelial cells and usually negative in adenocarcinoma, while MOC-31, Ber-EP4, B72.3 (BRST-3), and CEA are positive in adenocarcinoma and usually negative in mesothelial cells. Similarly, CEA, CK7, CK20, TTF1, and CDX2 are usually positive in adenocarcinoma cells, while WT-1, calretinin, D2-40, CK5/6, and cytokeratin are often positively expressed in mesothelioma cells.

Based on the good performance of ROSE in differentiating benign and malignant pleural biopsies, we believe that ROSE also contributes to the triage of samples for auxiliary detection, such as IHC, gene mutation detection, microbial culture, and molecular tests. For example, for biopsies that ROSE diagnosed as adenocarcinoma, thoracoscopists could appropriately obtain more specimens for genetic testing for targeted drug therapy. For biopsies diagnosed as TB, more samples can be obtained simultaneously for microbial culture, NAAT, and Z–N staining, avoiding a greater turnaround time. Collins et al. (43) concluded that ROSE can improve cell block quality and provide better utilization for IHC assessment and IHC testing in positive diagnostic category cases. Fetzer et al. (44) and Capková and Galgonkvá (45) reported that cytotechnologists or cytopathologists performed at a high level of competency in providing ROSE and allocating specimens for ancillary studies.

There are some limitations to our study. This is a retrospective cohort study with consecutive patients, and multicenter prospective observational studies or randomized multicenter studies are needed to eliminate bias in total pleural biopsies and ROSE diagnostic samples and to further evaluate the ability of ROSE to reduce the number of biopsies, shorten the procedure time, assess cost-effectiveness, and guide pleurodesis during MT. Additionally, this study was conducted in an area where TB is endemic, and the results of the ROSE interpretation of tuberculous pleural disease may not be generalizable to other areas. Importantly, ROSE specimens were interpreted by two cytopathologists (Dr. Wang and Dr. Li) with over 10 years of experience in cytopathology in our center, and they were blinded to the clinical history/data of the histopathologist who read the definitive histology; therefore, there is no possible bias in the sensitivity and specificity of ROSE. If the ROSE specimens are interpreted by a clinical pulmonologist or thoracic surgeon who knows the clinical history/data, the diagnostic performance of ROSE may be biased.

In conclusion, ROSE of touch imprints of MT biopsies during MT showed high accuracy for distinguishing between benign and malignant lesions. In addition, ROSE is in good agreement with histopathological diagnosis, which may help thoracoscopists perform pleurodesis (talc poudrage) directly during the procedure, especially in cases showing malignant pleural effusion.

## Data availability statement

The raw data supporting the conclusions of this article will be made available by the authors, without undue reservation.

## Ethics statement

Written informed consent was obtained from the individual(s) for the publication of any potentially identifiable images or data included in this article.

## Author contributions

HW: conceptualization (equal), data curation (lead), formal analysis (equal), investigation (lead), methodology (equal), resources (equal), software (equal), visualization (equal), writing—original draft (lead), and writing—review and editing (lead). YL: conceptualization (equal), data curation (equal), formal analysis (equal), resources (equal), and visualization (equal). JW: visualization (equal), writing—original draft (equal), and writing—review and editing (equal). TR: methodology (equal), resources (equal), validation (equal), and writing—review and editing (equal). GL: methodology (equal), project administration (equal), and resources (equal). HY: methodology (equal), project administration (equal), validation (equal), and visualization (equal). XW: software (equal) and validation (equal). DL: supervision (supporting), writing—original draft (supporting), and writing—review and editing (supporting). LW: project administration (equal) and resources. MW: conceptualization (equal), project administration (lead), and visualization (lead). All authors contributed to the article and approved the submitted version.
