# Supplementary material for: Rapid on-site evaluation of touch imprints of medical thoracoscopy biopsy tissue for the management of pleural disease
Source: Front Med (Lausanne). 2023 Jun 9;10:1196000. doi: 10.3389/fmed.2023.1196000 (PMC10288871; doi:10.3389/fmed.2023.1196000)
Supplement: Supplementary file 1 [file Data_Sheet_1.docx]

Supplemental table. Laboratory results of peripheral blood in enrolled patients (n=565)

| Laboratory tests | Group A  (n=183) | Group B  (n=382) | χ^2^/*t*/Z | *p* value |
| --- | --- | --- | --- | --- |
| Peripheral blood |  |  |  |  |
| Neutrophils,10^9^/L | 5.6(4.2,7.8) | 4.8 (3.6,6.2) | -9.9 | <0.01 |
| lymphocytes,10^9^/L**^*^** | 1.6±0.5 | 1.4±0.6 | 5.02 | 0.00 |
| monocytes,10^9^/L | 0.6(0.4,0.8) | 0.6 (0.5,0.9) | -1.2 | 0.23 |
| platelets,10^9^/L**^*^** | 292.6±99.9 | 329.8±98.5 | -78.5 | 0.00 |
| Fibrinogen, g/L**^*^** | 5.5±1.6 | 6.0±1.7 | -8.4 | 0.00 |
| D-Dimer, mg/L | 3.3(0.9,5.5) | 2.1(1.0,3.0) | -7.3 | <0.01 |

**^*^** *t* test used, others are Mann-Whitney U test.
